# Characterization of the Mitogenome of the Genus *Dendrocerus* Ratzeburg (Hymenoptera: Megaspilidae) with the Specific Designed Primers

**DOI:** 10.3390/ani14101454

**Published:** 2024-05-13

**Authors:** Xu Wang, Wenjing Zhao, Shanshan Cui, Baoshan Su, Yixin Huang, Huayan Chen

**Affiliations:** 1 Anhui Provincial Key Laboratory of the Conservation and Exploitation of Biological Resources, College of Life Sciences, Anhui Normal University, Wuhu 241000, China; wangxu0322@ahnu.edu.cn (X.W.); zhaowenjing2121@163.com (W.Z.); 18437995883@163.com (S.C.); 2 Key Laboratory of Zoological Systematics and Evolution, Institute of Zoology, Chinese Academy of Sciences, Beijing 100000, China; huangyx@ahnu.edu.cn; 3 Collaborative Innovation Center of Recovery and Reconstruction of Degraded Ecosystem in Wanjiang Basin Co-Founded by Anhui Province and Ministry of Education, School of Ecology and Environment, Anhui Normal University, Wuhu 241000, China; su13966489655@163.com; 4 Key Laboratory of Plant Resources Conservation and Sustainable Utilization, Chinese Academy of Sciences, Guangzhou 510650, China; 5 State Key Laboratory of Plant Diversity and Specialty Crops, South China Botanical Garden, Chinese Academy of Sciences, Guangzhou 510650, China; 6 South China National Botanical Garden, Guangzhou 510650, China

**Keywords:** Evaniomorpha, Ceraphronoidea, mitochondrial genome, pairwise breakpoint distance, phylogenetic tree

## Abstract

**Simple Summary:**

In this study, we sequenced two mitochondrial genomes of *Dendrocerus* (Hymenoptera: Megaspilidae) to analyze the mitochondrial genomic features of *Dendrocerus* and provide new molecular data for phylogenetic studies of Evaniomorpha. The phylogenetic results revealed that Evaniomorpha is not a monophyletic group, which is also supported by the PBD (Pairwise breakpoint distances) values. Additionally, Ceraphronoidea is a monophyletic group and is a sister to Aulacidae + Gasteruptiidae. Furthermore, specific primers MegaF/MegaR were designed for Megaspilidae based on the “conserved regions” of *COX1* sequences. They exhibited a good amplification efficiency of 60% for the genus *Dendrocerus.* This study provides new molecular data for phylogenetic studies of Evaniomorpha, further offering the possibility of solving a controversial problem in the phylogeny of Evaniomorpha and providing a solution to the problem of difficult amplification of the *COX1* gene in Ceraphronoidea.

**Abstract:**

In Hymenoptera, the monophyly of Evaniomorpha has been the focus of debate among different scholars. In this study, we sequenced two mitochondrial genomes of *Dendrocerus* (Hymenoptera: Megaspilidae) to analyze the mitochondrial genomic features of *Dendrocerus* and provide new molecular data for phylogenetic studies of Evaniomorpha. The mitogenome sizes of *D. bellus* and *D. anisodontus* were 15,445 bp and 15,373 bp, respectively, with the *trnG* of *D. bellus* missing. The nucleotide composition was significantly biased toward adenine and thymine, with A + T contents of 81.2% (*D. bellus*) and 82.4% (*D. anisodontus*). Using *Ceraphron* sp. (Ceraphronidae) as reference, the Ka/Ks values of *NAD4L* and *NAD6* in *D. anisodontus* were both greater than one, indicating that non-synonymous mutations are favored by Darwinian selection, which is rare in other hymenopteran species. Compared with *Ceraphon* sp. gene order, nine operations were identified in *D. anisodontus*, including four reversals, four TDRLs (tandem duplication random losses) and one transposition, or four reversals and five TDRLs. Phylogenetic analysis of 40 mitochondrial genomes showed that Evaniomorpha was not a monophyletic group, which was also supported by the PBD values. Ceraphronoidea is a monophyletic group and is a sister to Aulacidae + Gasteruptiidae. Based on the conserved region of the newly sequenced mitochondrial genomes, a pair of specific primers MegaF/MegaR was designed for sequencing the *COX1* genes in Megaspilidae and a 60% rate of success was achieved in the genus *Dendrocerus*.

## 1. Introduction

Megaspilidae is a small but widespread family of parasitoid wasps that contains approximately 450 species in 12 genera [1,2]. The monophyly of Evaniomorpha, to which the Megaspilidae belong, has been the focus of much scrutiny over the last decade, based on either morphological or molecular data, or both [3,4,5,6]. However, within Evaniomorpha, there is little consensus on the phylogenetic relationships among the superfamilies, due to the lack of reliable morphological characters to delimit the superfamilies and the limited molecular markers for constructing a robust phylogeny [7,8,9,10]. Mitochondrial (mt) genomes are one of the most highly studied genomic systems for species identification, molecular evolution, and phylogenetic inference [11,12,13,14]. In Hymenoptera, mitochondrial genomes have been extensively sequenced and provide useful genetic markers for phylogenetic inferences [13,14,15,16]. However, compared to other groups of Hymenoptera, the use of mitochondrial genomes for the phylogenetic analyses in Megaspilidae and Evaniomorpha is still scarce.

The taxonomy of Megaspilidae depends heavily on the morphology of the male genitalia [17]. Although effective in species delimitation, species identification by male genitalia has limitations, such as failure in matching the two sexes of the same species. Combining molecular identification can address this issue, but there are challenges in obtaining DNA barcodes (*COX1*) in Megaspilidae using universal primers due to the small size of the parasitoid, resulting in reduced amounts of extracted DNA and mixed DNA originating from the parasitioid and its host [18]. Vasilita et al. (2022) [18] developed an optimized barcoding protocol for Ceraphronoidea, but the success rate of barcoding dropped as more samples were tested, and its applicability in Megaspilidae is not clear. This suggests that new specific barcode primers designed with reference to complete mitochondrial genomes may be required.

*Dendrocerus* Ratzeburg, 1852, is the most representative and economically important genus of Megaspilidae [19]. It covers the range of all trophic levels from primary to quaternary parasitoids and has been used as a model system for understanding the ecology of parasitoidism [20]. In a previous study, we reported two species of *Dendrocerus* from China, *Dendrocerus bellus* Wang, Chen & Mikó, and *Dendrocerus anisodontus* Wang, Chen & Mikó [21]. Based on antennal characters, *D. bellus* and *D. anisodontus* belong to two representative species groups, *halidayi* and *arpenter*, respectively. Therefore, in this study, we sequenced two mitochondrial genomes of both species (*Dendrocerus bellus* Wang, Chen & Mikó and *Dendrocerus anisodontus* Wang, Chen & Mikó) and analyzed their characterizations. We then explored the phylogenetic relationships of Evaniomorpha based on the analyses of 38 hymenopteran mitogenomes downloaded from GenBank and the two newly generated sequences of Megaspilidae, with the aim of providing possibilities for solving controversial issues in the phylogeny of Evaniomorpha. Furthermore, based on the “conserved regions” of *COX1* sequences, we designed specific primers for Megaspilidae, aiming to provide a solution to the difficult amplification of the *COX1* gene in Ceraphronoidea.

## 2. Materials and Methods

### 2.1. Sample Preparation and DNA Extraction

All specimens of *D. bellus* and *D. anisodontus* were collected from the wild in Guangzhou, Guangdong, China, using sweep nets, Malaise traps, and yellow pan traps. All the specimens were stored at −20 °C in absolute ethanol prior to the selection of one sample of each species for DNA extraction and sequencing. Total genomic DNA was extracted using the cetyltrimethyl ammonium bromide (CTAB) method [22].

### 2.2. Sequencing and Assembly

Sequencing was performed using a whole genome shotgun (WGS) strategy on the Illumina Miseq platform. The quality of data was checked with FastQC [23,24]. The original data adapters were removed by AdapterRemoval version 2 [25]. SOAPec version 2.01 was used for quality correction with the K-mer set to 17 [24]. Reads with a length of less than 50 bp were excluded. A5-miseq version 2.0 was used to complete the assembly of the mitochondrial (mt) genome [24,26].

### 2.3. Mitochondrial Genome Annotation

The tRNA genes and protein-coding genes were identified using MITOS WebSever. The secondary structure was also predicted by the MITOS WebSever, with parameters set to the Invertebrate Mito genetic code [24,27]. Protein-coding genes (PCGs) were identified as open reading frames corresponding to the 13 PCGs in the metazoan mitogenomes and were checked manually. The mitogenome maps were produced using Organellar Genome DRAW (OGDRAW) [28].

### 2.4. Mitochondrial Genome Comparative Analysis

Base composition and relative synonymous codon usage (RSCU) were analyzed using MEGA X [29]. All the genes in the mitochondrial genome were checked using Geneious 10.0.5 (Biomatters, Auckland, New Zealand). Nucleotide composition, codon usage, comparative mitogenomic architecture for these two mitogenomes, and data used to plot RSCU (relative synonymous codon usage) were all calculated using PhyloSuite [27,30]. The predicted secondary structures of all tRNAs were drawn by Adobe Illustrator CC 2018 according to the MITOS predictions. Non-synonymous (Ka)/synonymous (Ks) mutation rate ratios among the 13 PCGs were calculated with DnaSP v5 [31,32]. AT/GC skewness was calculated as AT skew = (A − T)/(A + T) and GC skew = (G − C)/(G + C) [33]. A value of 0 means a balance between the bases in the two chains and no deviation in the base composition.

### 2.5. Phylogenetic Analysis

In this study, a dataset comprised 38 mitogenomes from GenBank, and two newly generated sequences were used for phylogenetic analysis. *Labriocimbex sinicus* (Cimbicidae) and *Praia tianmunica* (Cimbicidae) were used as the outgroups to reconstruct the phylogenetic tree of Apocrita. Each PCG was aligned individually based on codon optimized multiple alignments using MAFFT 7.3.1 with G-INS-I algorithms [34]. The aligned sequences were then concatenated and split into datasets (PCG12: including 13 PCGs without the third codon sites). Maximum likelihood (ML) analysis was conducted on the concatenated dataset for phylogeny reconstruction implemented in W-IQtree using the best-fit substitution model [35]. An ultrafast bootstrap (UFB) [36] of 1000 replications and the SH-aLRT test were used in this analysis to assess branch supports [37]. The resulting trees were visualized using FigTree v.1.3.1 and embellished with the Adobe Photoshop CS3 software.

Pairwise breakpoint distances (PBDs) between the mitochondrial genomes of each species were calculated using the web server CREx to analyze the proximity of species to each other, and heatmaps were constructed with eGPS 64 bit.

### 2.6. Specific Primer Design

Specific primers were designed based on the mitochondrial genome sequences of *D. bellus* and *D. anisodontus*, using the PrimerPremier 5 software to locate the “conserved regions”. The primer information was as follows: MegaF, 5′-ATAGAACTAATCACAAATTTATTGG-3′ (157 bp at the 5′ end position, Tm = 47.8 °C), and MegaR, 5′-TAAACTTCAGGGTGACCAAAGAATCA′ (389 bp at the 3′ end position, Tm = 54.8 °C). The primer amplification efficiency test included 15 samples of Megaspilidae, collected in Chongqing, China. Non-destructive DNA extraction was performed using the TIANamp Genomic DNA Kit. The DNA quantity in the eluate was checked prior to PCR reactions using an Implen NanoPhotometer N60. Amplification of the mitochondrial *COX1* was conducted in a 20 µL PCR reaction with 2 µL DNA template. The cycling conditions were set as follows: pre-denaturation at 94 °C for 5 min, followed by 35 cycles of denaturation at 94 °C for 1 min, annealing at 51 °C for 1 min, and extension at 72 °C for 1 min. After the cycling, a final extension was performed at 72 °C for 5 min.

## 3. Results

### 3.1. Mitogenome Structure and Organization

The mitogenome lengths of *D. bellus* and *D. anisodontus* are 15,445 bp and 15,373 bp, respectively, with the trnG of *D. bellus* missing. *D. anisodontus* mainly contains 22 tRNA genes, 2 rRNA genes (rrnL and rrnS), and 13 PCGs (*NAD1*, *NAD2*, *NAD3*, *NAD4*, *NAD5*, *NAD6*, *NAD4L*, *COX1–3*, *ATP8*, *ATP6*, and *CTYB*). *D. bellus* mainly contains 21 tRNA genes, 2 rRNA genes (*rrnL* and *rrnS*), and 13 PCGs (*NAD1*, *NAD2*, *NAD3*, *NAD4*, *NAD5*, *NAD6*, *NAD4L*, *COX1–3*, *ATP8*, *ATP6*, and *CTYB*).

**Figure 1:**
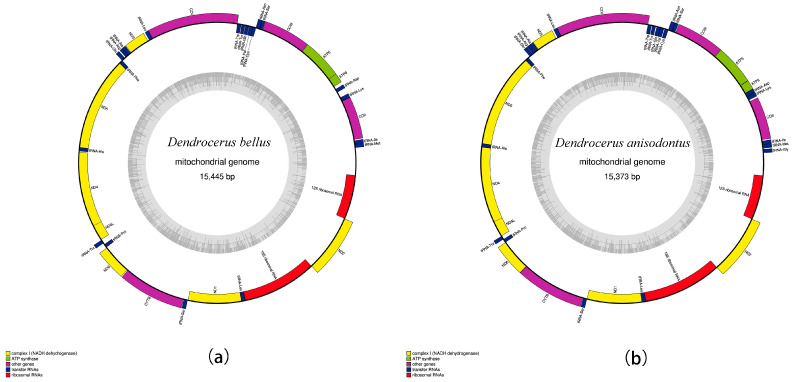
Circular maps of the mitochondrial genome: (**a**) *Dendrocerus bellus* and (**b**) *Dendrocerus anisodontus.* Protein-coding and ribosomal genes are indicated using standard abbreviations. The J-strand is shown on the outer circle and the N-strand on the inner circle.

The transcription direction of the encoding genes in the genomes of these two species is generally consistent with that of other insects. Among the two species, 21 genes (*trnM*, *trnI*, *COX2*, *trnK*, *trnD*, *ATP6*, *ATP8*, *COX3*, *trnS*, *trnN*, *COX1*, *trnL*, *NAD3*, *trnA*, *trnR*, *trnE*, *trnT*, *NAD6*, *CYTB*, *trnS*, and *NAD2*) were located in the J chain, and 14 genes (*trnC*, *trnV*, *trnQ*, *trnY*, *trnW*, *trnF*, *NAD5*, *trnH*, *NAD4*, *NAD4L*, *trnP*, *NAD1*, *trnL*, and *rrnL*) were located in the N chain, While the *rrnS* of *D. bellus* is located on the N chain, and the *trnG* and *rrnS* of *D. anisodontus* are located on the J chain (Figure 1).

### 3.2. Protein-Coding Genes

The size of 13 PCGs were 11,173 bp and 11,214 bp, respectively (Table 1), accounting for 72.34% and 72.95% of the entire genome, respectively. The average AT content of the 13 protein-coding genes in *D. anisodontus* was 80.90% and 79.20% in *D. bellus*. The 13 PCGs with the lowest single AT content were all *COX1*, with 70.50% in *D. bellus* and 72.90% in *D. anisodontus*; the 13 PCGs with the highest single AT content were all *NAD2*, with 87.00% in *D. bellus* and 87.30% in *D. anisodontus* (Table 2).

All 13 protein-coding genes (PCGs) were initiated with the standard start codon of ATN. Four types of start codons—ATA, ATT, ATG, and ATC—were used (Table 2). Additionally, there were three types of stop codons: TAA, TAG, and T. The *COX2*, *ATP8*, *ATP6*, *COX3*, *NAD3*, *NAD4L*, and *NAD2* genes were stopped with TAA in the two species. The *COX1*, *NAD4*, and *NAD6* genes in *D. bellus* and the *CYTB* and *NAD1* genes in *D. anisodontus* were also stopped with TAA. The *CYTB* and *NAD1* genes in *D. anisodontus* were stopped with TAA and the other genes had an incomplete stop codon T (Table 2). Incomplete termination codons are common in animal mitochondrial DNA and are likely to be completed by post-transcriptional polyadenylation [38].

**Figure 2:**
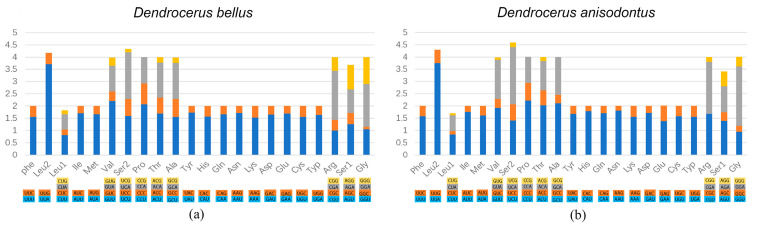
Relative synonymous codon usage (RSCU) of the mitochondrial genomes of (**a**) *Dendrocerus bellus* and (**b**) *Dendrocerus anisodontus*.

Codons with high A/T content were preferred in these two species, as in most insect mitochondrial genomes [39]. In the two studied species, Ala, Gly, Leu, Pro, Arg, Ser, Thr, and Val were the most frequently used amino acids, and UUA (Leu) had the highest relative synonymous codon usage (RSCU) (Figure 2). The third codon position of A/T occurred more frequently than that of G/C, reflecting AT nucleotide bias in the mitochondrial PCGs among Megaspilidae.

### 3.3. Transfer RNA and Ribosomal RNA Genes

**Figure 3:**
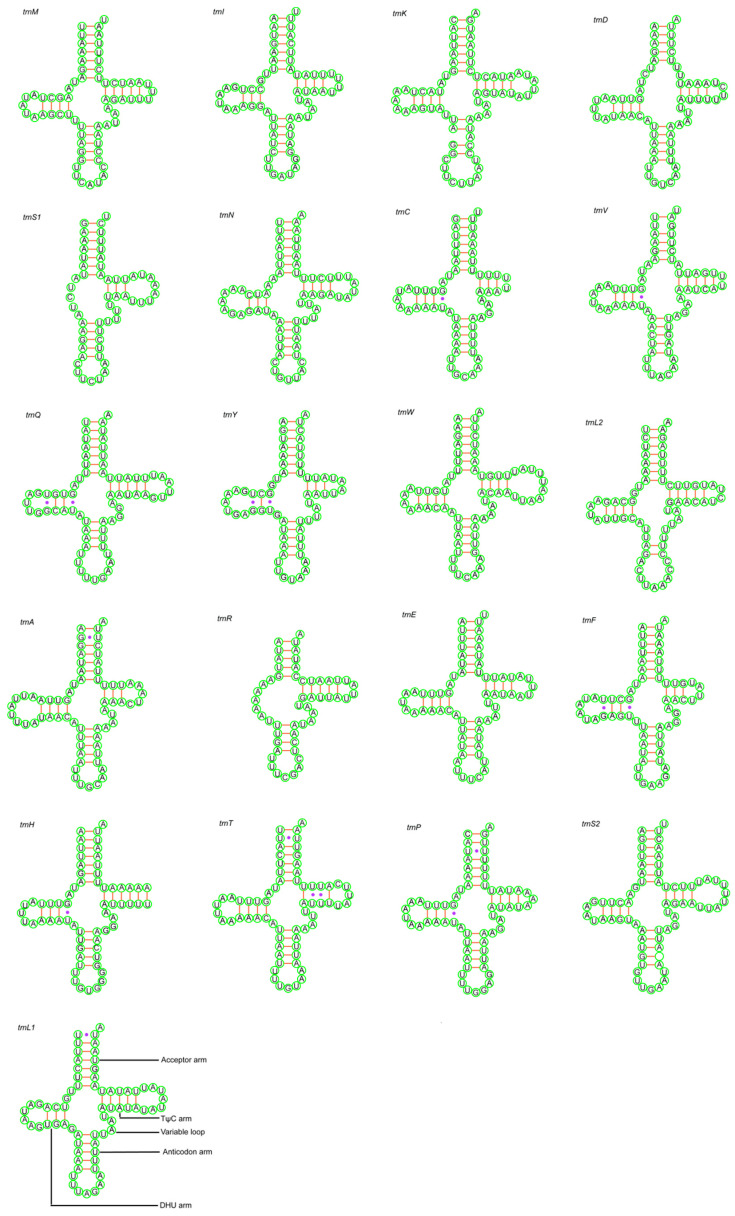
Putative secondary structures of tRNAs from the *Dendrocerus bellus* mitogenome. Purple dots indicate base mismatches.

**Figure 4:**
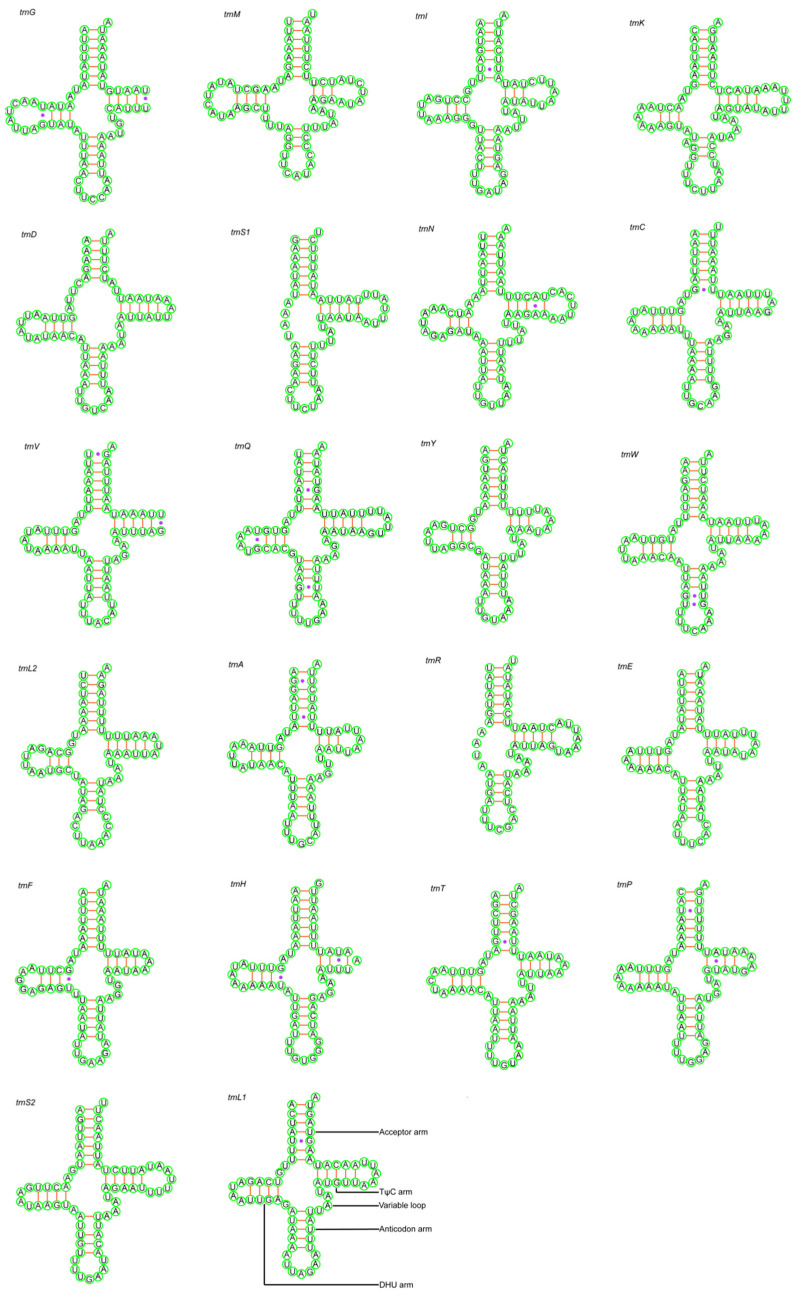
Putative secondary structures of tRNAs from the *Dendrocerus anisodontus* mitogenome. Purple dots indicate base mismatches.

In total, 21 tRNAs of *D. bellus* and 22 tRNAs of *D. anisodontus* genes were interspersed throughout the two Megaspilidae mitochondrial genomes. These two species had similar features in the tRNA and rRNA genes. The tRNA genes of the two mitogenomes were dispersed among the genes of rRNA and PCG. Most tRNAs could be folded into the clover-leaf secondary structures, while the trnS1 and trnR of both species lack the DHU arm (Figure 3 and Figure 4). The tRNAs of the two species range from 52 to 69 bp and 56 to 70 bp, respectively (Table 2). Their positions and sizes follow the typical organization for insect mtDNA.

In these two species, the length of rrnS is 785 bp; the length of rrnL of *D. anisodontus* is slightly longer than that of *D. bellus*, 1384 bp and 1336 bp, respectively. For the AT contents of *rrnL*, the content of *D. anisodontus* is slightly higher than that of *D. bellus*, with 86.9% and 86.7%, respectively. The AT content of *rrnS* is slightly higher in *D. bellus* than in *D. anisodontus*, at 87.4% and 86.5%, respectively.

### 3.4. Overlap and Gap

The number of gap sites and bases as well as the number of overlap sites and bases in *D. bellus* were all slightly higher than those in *D. anisodontus*. Among the 21 gap sites of *D. bellus*, there were a total of 343 bp intergenic nucleotides, ranging from 1 bp to 105 bp. The longest gap between *trnK* and *trnD* was 105 bp. The shortest gap was 1 bp, which was located between *COX2* and *trnK* and *NAD5* and *trnH*. *D. anisodontus* had 17 gap sites, and a total of 196 bp, ranging from 1 bp to 34 bp. The longest gap was 34 bp between *trnE* and *trnF*, and the shortest gap was 1 bp, located between *trnV* and *trnQ* and between *trnP* and *trnT*.

Among the 7 gaps of *D. bellus*, there were a total of 22 bp of overlapping nucleotides. *D. anisodontus* had 5 overlapping sites totaling 16 bp. The overlapping sites in both species ranged from 1 bp to 7 bp. The longest gap between *NAD4* and *NAD4L* was 7 bp, and the shortest gap was 1 bp. They differed due to the fact that the shortest gap in *D. bellus* was between *trnA* and *trnR* or *trnE* and *trnF*, while the shortest gap in *D. anisodontus* was between *trnK* and *trnD* (Table 1).

### 3.5. Evolutionary Rate Analysis

**Figure 5:**
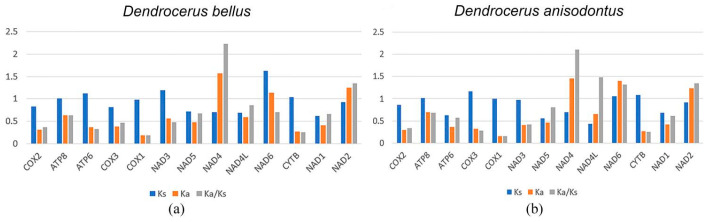
Evolutionary rates of mitochondrial genomes. The numbers of non-synonymous substitutions per non-synonymous site (Ka), the number of substitutions per synonymous site (Ks), and the ratio of Ka/Ks for every mitochondrial gene are given, using *Ceraphron* sp. as the reference sequence. (**a**) *Dendrocerus bellus* and (**b**) *Dendrocerus anisodontus*.

The estimated evolutionary rates for non-synonymous and synonymous substitution across the 13 PCGs in the two mitochondrial genomes range from 0.1592 to 2.2323. In the two species, the largest Ka/Ks values are in *NAD4*, which are 2.2323 and 2.1057; the smallest Ka/Ks values are in *COX1*, which are 0.1838 and 0.1592, respectively (Figure 5).

In both species, the highest Ka/Ks values are *NAD4*, which are 2.2323 and 2.1057, respectively; the Ka/Ks values of *NAD2* are both greater than 1, which are 1.3502 and 1.3417, respectively. In *D.anisodontus*, the Ka/Ks values of *NAD4L* and *NAD6* are both greater than one, indicating that non-synonymous mutations are favored by Darwinian selection, and they will be retained at a rate greater than synonymous mutations, which is rare in other species. Otherwise, most of the 13 protein-coding genes in the two species were purified and selected.

### 3.6. Gene Arrangement

**Figure 6:**
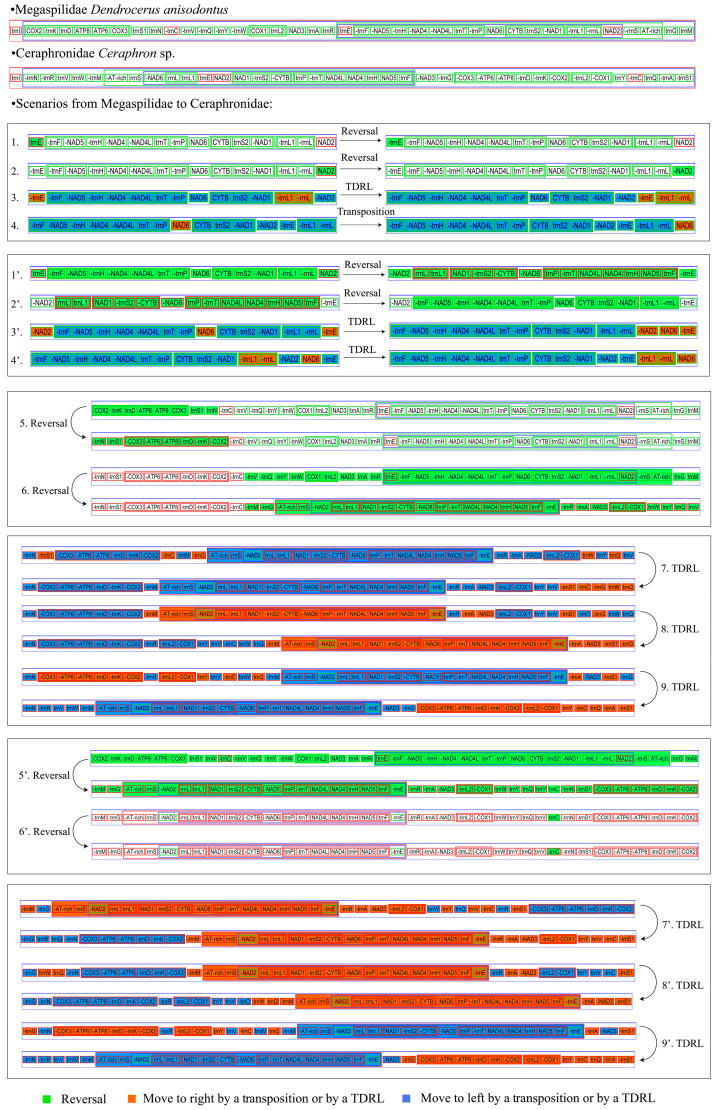
Mitochondrial gene order rearrangement scenario inferred by CREx for the given phylogeny of Megaspilidae. Green color indicates: reversal. Red color indicates: move to right by a transposition or by a TDRL. Bule color indicates: move to left by a transposition or by a TDRL. From step one to step four there are two programs 1-4 and 1’-4’; from step five to step nine there are two programs 5-9 and 5’-9’.

We inferred the evolution of gene arrangement in Megaspilidae using CREx by comparing the common intervals between Megaspilidae and Ceraphronidae gene order (Figure 6) [40]. Three operations were considered in CREx, i.e., transposition, reversal, and TDRL. From Megaspilidae *D. anisodontus* gene order to Ceraphronidae *Ceraphon* sp. gene order, the CREx identified nine operations, including four reversal (operations 1–2 and 5–6 in Figure 6), four TDRLs (operations 3 and 7–9 in Figure 6), and one transposition (operation 4 in Figure 6, referring to *NAD6*) or four reversals (operations 1′–2′ and 5′–6′ in Figure 6) and five TDRLs (operations 3′–4′ and 7′–9′).

There are two sets of alternative scenarios in operations 1–4. The first set of scenarios refers to two reversals of *trnE* and *NAD6*, followed by one TDRL and one transposition, while the other set refers to two reversal and two TDRLs. There are also two optional scenarios in operations 5–9. In the first scenario of operations 5–9, the first step is one reversal from *COX2-trnK-trnD-ATP8-ATP6-COX3-trnS1-trnN* to *trnN-trnS1-COX3-ATP6-ATP8-trnD-trnK-COX2*; the second step is one reversal of nine protein genes, 17 tRNA genes, and two rRNA genes; the remaining steps are three TDRLs. The second set of scenarios in operations five–nine also refers to two reversals and three TDRLs.

### 3.7. Phylogenetic Analysis

**Figure 7:**
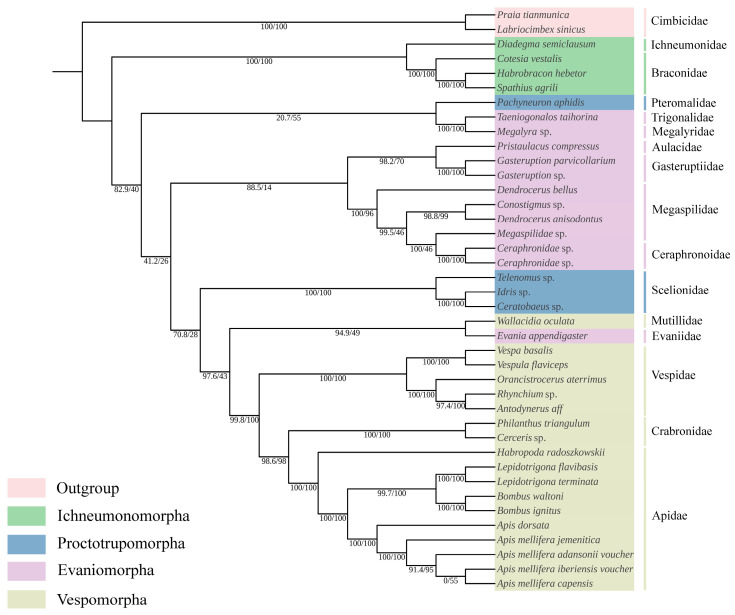
ML phylogenetic trees of Apocrita based on PCG12 matrix.

Based on the matrix of PCG12, a robust phylogenetic tree was achieved with high bootstrap values or posterior probabilities (Figure 7). Ceraphronoidea is clearly a monophyletic group and forms a sister group with (Aulacidae + Gasteruptiidae). Evanioidea (Evaniidae, Aulacidae and Gasteruptiidae) are also not a monophyletic group, as Evaniidae is a sister group of Mutiiidae. The relationship between Pteromalidae, Megalyridae, and Trigonalyidae is as follows: (Pteromalidae + (Megalyridae + Trigonalyidae)). Therefore, Evaniomorpha is not a monophyletic group.

**Figure 8:**
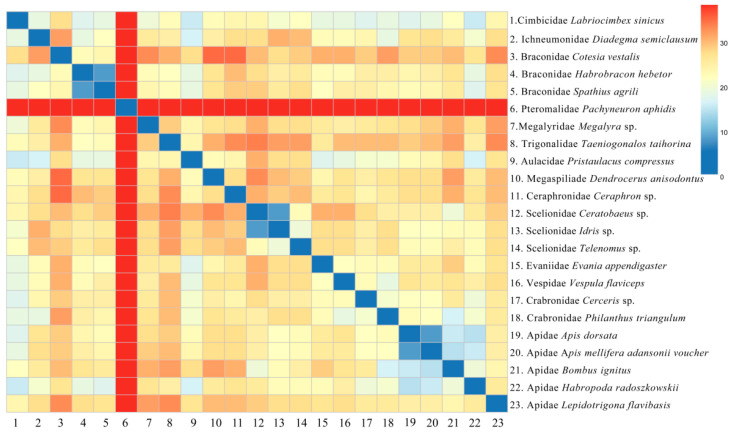
The heat map of pairwise breakpoint distance of Apocrita.

The value of pairwise breakpoint distances between Aulacidae, *Pristaulacus compressus,* and Evaniidae, *Evania appendigaster,* was 12. For Aulacidae, *Pristaulacus compressus,* and Vespidae, *Vespula flaviceps,* the value was 13. Similarly, for Aulacidae, *Pristaulacus compressus,* and Apidae, *Habropoda radoszkowskii,* the value was 11 (Figure 8). Lower values of PBD indicated closer relationships which are not consistent with the topology among Aulacidae, Evaniidae, Vespidae, and Apidae on the phylogenetic tree. The PBD values of Pteromalidae, *Pachyneuron aphidis,* and other species were greater than 34, which are also inconsistent with the phylogenetic tree. Evaniomorpha is not a monophyletic group according to the phylogenetic relationships shown by the PBD values, which is consistent with the phylogenetic tree.

### 3.8. Specific Primer Design

Based on the conserved regions of Megaspilidae *COX1* sequences, we designed a pair of specific primers. i.e., MegaF, 5′-ATAGAACTAATCACAAATTTATTGG-3′ (5′ end position 157 bp, Tm = 47. 8 °C); MegaR, 5′-TAAACTTCAGGGTGACCAAAGAATCA′ (3′ end position 389 bp, Tm = 54.8 °C), with the production size of 650 bp. The newly designed primers were tested for efficiency with 15 specimens of *Dendrocerus*, and the amplification success rate was 60% (Table 3 and Table 4).

## 4. Discussion

Currently, only two complete mitochondrial genomes of Megaspilidae are available, and no mitogenomes of *Dendrocerus* species have been reported in the NCBI database. In this study, we first sequenced and analyzed two mitochondrial genomes from *Dendrocerus* (*D. bellus* and *D. anisodontus*). Like other parasitic wasps, the *Dendrocerus* had a high A + T content and a negative GC skew. However, in the present study, *D. bellus* and *D. anisodontus* had opposite AT skew of −0.0025 and 0.0243, respectively, which might be due to the deletion of the *trnG* gene in *D. bellus.*

We estimated the average Ka/Ks values for each PCG to better understand the function of selection pressure and the development of the two mitochondrial genomes. Among the 13 PCGs of *D. bellus* and *D. anisodontus*, *NAD4* had the highest average Ka/Ks of 2.2323 and 2.1057, and *COX1* had the lowest average Ka/Ks of 0.1838 and 0.1592, respectively. In *D. anisodontus*, the Ka/Ks values of *NAD4L* and *NAD6* were both greater than one, indicating that non-synonymous mutations are favored by Darwinian selection, and they will be retained at a rate greater than synonymous mutations, which is rare in other species.

Codon usage bias can be an indicator of the selective pressure operating at the molecular level [41]. The relative synonymous codon usage (RSCU) rates and codon distributions for the two mitochondrial genomes (Figure 2) showed that the most frequently used amino acids were Ala, Gly, Leu, Pro, Arg, Ser, Thr, and Val, and the highest relative synonymous codon usage was UAA. In addition, the codons were biased to utilize more A/U than G/C, which resulted in the AT content being higher than GC in the PCGs.

The mitogenome gene order of *Dendrocerus* is consistent with *Conostigmus* without gene rearrangement, indicating that the gene arrangement within Megaspilidae might be conserved. However, due to the lack of mitogenome data from other genera of Megaspilidae, this conclusion needs further investigation. A series of gene rearrangements are evident relative to the putative ancestral pancrustacean mitochondrial genome. Compared with the ancestor *Drosophila yakuba* (Diptera, Drosophilidae), 11 tRNA and two protein genes were rearranged in Megaspilidae [42]. The most striking gene rearrangement in the Megaspilidae resulted from the separation of two rRNA genes (*rrnL*, *rrnS*) by the protein-coding genes *NAD2* (*NAD6* in the Ceraphronidae), which was not observed in other Hymenoptera [8]. The mitogenomes gene order of Megasoplidae has changed considerably compared to Ceraphonidae. Therefore, we inferred the evolution of gene arrangement in Megaspilidae using CREx by comparing the common intervals between Megaspilidae and Ceraphronidae gene orders. From Megaspilidae gene order to Ceraphronidae gene order, there are two rearrangement pathway, including four reversal, four TDRLs, and one transposition or four reversals and five TDRLs.

To date, most molecular phylogenies involving Evaniomorpha were dependent on relatively short mt and nuclear gene fragments [8]. Few studies had used the entire mt genome and pairwise breakpoint distances (PBDs) to assess phylogenies of Evaniomorpha or higher-level taxa in Apocrita. Our study is the first attempt to use PBD to analyze the phylogeny of Evaniomorpha. The PBD value between *Pristaulacus compressus* (Aulacidae) and *Habropoda radoszkowskii* (Apidae) was 11, while the PBD value between *Pristaulacus compressus* (Aulacidae) and *Diadegma semiclausum* (Ichneumonidae) was also 11. The PBD value between *Pristaulacus compressus* (Aulacidae) and *Megalyra* sp. (Megalyridae) was 18, and the PBD value between *Pristaulacus compressus* (Aulacidae) and *Taeniogonalos taihorina* (Trigonalidae) was 18. Lower PBD values indicate that Aulacidae *Pristaulacus compressus* was more closely related to *Habropoda radoszkowskii* (Apidae) and *Diadegma semiclausum* (Ichneumonidae), which was not consistent with the phylogenetic tree, but indirectly suggests the non-monophyly of Evaniomorpha. Ceraphronoidea (Megaspilidae and Ceraphronoidae) are robustly monophyletic, which was confirmed in our study [17]. However, the phylogenetic placement of Ceraphronoidea within Apocrita remains unresolved, with current molecular studies indicating a position as a sister group to Ichneumonoidea or as sister group to Evaniidae, in a clade that is a sister group to Aculeata or as a sister group to (Evanioidea + Stephanoidea) [6,9]. In our study, the Ceraphronoidea was related to (Aulacidae + Gasteruptiidae) as a sister group, which was inconsistent with the conclusion of previous studies. Based on ultraconserved elements (UCEs) and mitochondrial genomes, respectively, Blaimer et al. (2023) and Mao et al. (2014) [8] considered Megalyridae and Trigonalidae form a sister group, which is further confirmed in our study.

Our study presents some hypotheses for the phylogenetic relationships of Evaniomorpha, but many questions remain unresolved. In particular, there is still considerable uncertainty regarding the relationships within Evaniomorpha and the nested relationships between Evaniomorpha and Aculeata. More mitochondrial genome data are required to provide sufficient evidence for understanding the phylogenetic relationships of Evaniomorpha in the future.

The newly designed primer pair MegaF/MegaR worked well for *Dendrocerus*. However, due to the limited diversity of samples tested so far, further studies on Megaspilidae specific primers are still needed to assess the amplification efficiency in other genera of Megaspilidae.

## 5. Conclusions

By sequencing two mitochondrial genomes of *Dendrocerus* (Hymenoptera: Megaspilidae) and comprehensively analyzing multiple mitochondrial groups, we have arrived at the following conclusions.

The mitogenome sizes of *D. bellus* and *D. anisodontus* which are 15,445 bp and 15,373 bp, respectively, display a nucleotide composition heavily biased towards adenine and thymine. Notably, the Ka/Ks values of *NAD4L* and *NAD6* in *D. anisodontus* exceed one, suggesting a preference for non-synonymous mutations under Darwinian selection, which is uncommon in hymenopteran species. Moreover, *D. anisodontus* underwent nine rearrangements compared to *Ceraphon* sp., including four reversals, four tandem duplication random losses (TDRLs), and one transposition. Meanwhile, Ceraphronoidea is a monophyletic group that forms a sister group with (Aulacidae + Gasteruptiidae), while Evaniomorpha is not a monophyletic group, and this was further confirmed by congruence with PBD values. Additionally, utilizing the “conserved regions” of *COX1* sequences, we designed specific primers MegaF/MegaR tailored for Megaspilidae. These primers exhibited a satisfactory amplification efficiency of 60% for the *Dendrocerus* genus.

## Figures and Tables

**Table 1:** Nucleotide composition and skewness of mitogenomes.

Regions	Species	Size (bp)	T%	C%	A%	G%	AT (%)	GC (%)	AT Skew	GC Skew
Full genome	*Dendrocerus bellus*	15,445	40.7	12.2	40.5	6.7	81.2	18.8	−0.0025	−0.2910
	*Dendrocerus anisodontus*	15,373	40.2	11.7	42.2	5.9	82.4	17.6	0.0243	−0.3295
PCGs	*Dendrocerus bellus*	11,173	39.5	13.3	39.7	7.6	79.2	20.8	0.0025	−0.2727
	*Dendrocerus anisodontus*	11,214	39.5	12.6	41.4	6.6	80.9	19.1	0.0235	−0.3125
tRNAs	*Dendrocerus bellus*	1349	43.5	9.3	41.6	5.6	85.1	14.9	−0.0223	−0.2483
	*Dendrocerus anisodontus*	1415	43.1	9.2	42.3	5.4	85.4	14.6	−0.0094	−0.2603
rRNAs	*Dendrocerus bellus*	2121	43.8	9.1	43.1	3.9	86.9	13.1	−0.0081	−0.4000
	*Dendrocerus anisodontus*	2169	41.7	9.4	45.1	3.8	86.8	13.2	0.0392	−0.4242

**Table 2:** Mitogenomic organization of *D. bellus* and *D. anisodontus*.

	Position		Size (bp)		Intergenic Nucleotide	Codon	Strand
	From	To			Start	Stop	
*Dendrocerus bellus*/*Dendrocerus anisodontus*							
*trnG*	−/173	−/237	−/65				/+
*trnM*	264/253	328/320	65/68	−/15			+/+
*trnI*	326/318	389/382	64/65	−3/−3			+/+
*COX2*	410/402	1114/1103	705/702	20/19	ATT/ATT	TAA/TAA	+/+
*trnK*	1116/1132	1184/1200	69/69	1/28			+/+
*trnD*	1290/1200	1353/1265	64/66	105/−1			+/+
*ATP8*	1407/1266	1568/1424	162/159	53/−	ATC/ATA	TAA/TAA	+/+
*ATP6*	1565/1425	2242/2096	678/672	−4/−	ATA/ATG	TAA/TAA	+/+
*COX3*	2246/2100	3043/2897	798/798	3/3	ATG/ATG	TAA/TAA	+/+
*trnS1*	3047/2907	3101/2963	55/57	3/9			+/+
*trnN*	3102/2964	3168/3033	67/70	−/−			+/+
*trnC*	3166/3031	3225/3095	60/65	−3/−3			−/−
*trnV*	3223/3108	3287/3171	65/64	−3/11			−/−
*trnQ*	3292/3173	3359/3239	68/67	4/1			−/−
*trnY*	3365/3256	3426/3317	62/62	5/16			−/−
*trnW*	3436/3330	3594/3395	69/66	9/12			−/−
*COX1*	3505/3396	5052/4938	1548/1543	−/−	ATG/ATG	TAA/T	+/+
*trnL2*	5055/4939	5119/5002	65/64	2/−			+/+
*NAD3*	5147/5032	5509/5394	363/363	27/29	ATT/ATT	TAA/TAA	+/+
*trnA*	5517/5398	5582/5460	66/63	7/3			+/+
*trnR*	5582/5463	5633/5518	52/56	−1/2			+/+
*trnE*	5655/5522	5719/5584	65/63	21/3			+/+
*trnF*	5719/5619	5781/5681	63/63	−1/34			−/−
*NAD5*	5782/5682	7453/7350	1672/1669	−/−	ATT/ATT	T/T	−/−
*trnH*	7455/7351	7518/7411	64/61	1/−			−/−
*NAD4*	7531/7412	8868/8749	1338/1338	12/−	ATG/ATG	TAA/TAG	−/−
*NAD4L*	8862/8743	9143/9024	282/282	−7/−7	ATG/ATA	TAA/TAA	−/−
*trnT*	9152/9029	9217/9091	66/63	8/4			+/+
*trnP*	9244/9093	9307/9156	64/64	26/1			−/−
NAD6	9317/9157	9844/9706	528/550	9/−	ATC/ATA	TAA/T	+/+
*CYTB*	9860/9707	10,978/10,840	1119/1134	15/−	ATG/ATG	TAG/TAA	+/+
*trnS2*	10,986/10,847	11,053/10,914	68/68	7/6			+/+
*NAD1*	11,059/10,913	12,015/11,872	957/960	5/−2	ATA/ATA	TAG/TAA	−/−
*trnL1*	12,016/11,873	12,083/11,938	68/66	−/−			−/−
*rrnL*	12,084/11,939	13,419/13,322	1336/1384	−/−			−/−
*NAD2*	13,420/13,323	14,442/14,366	1023/1044	−/−	ATT/ATA	TAA/TAA	+/+
*rrnS*	14,443/14,367	15,227/15,151	785/785	−/−			−/+

**Table 3:** Detailed information of sequenced samples and accession numbers.

Sequencing Sample Number	Species	Sex	Location	GenBank Accession Number
A001	*D.* sp.1	female	Chongqing, Yintiaoling National Nature Reserve	OR578603
A002	*D.* sp.1	female	Chongqing, Yintiaoling National Nature Reserve	OR578604
A003	*D.* sp.1	male	Chongqing, Yintiaoling National Nature Reserve	OR578605
A004	*D.* sp.1	female	Chongqing, Yintiaoling National Nature Reserve	OR578606
A005	*D.* sp.1	female	Chongqing, Yintiaoling National Nature Reserve	OR578607
A006	*D.* sp.1	male	Chongqing, Yintiaoling National Nature Reserve	OR578608
A007	*D.* sp.2	male	Chongqing, Yintiaoling National Nature Reserve	OR578609
A008	*D.* sp.3	female	Chongqing, Yintiaoling National Nature Reserve	OR578610
A009	*D.* sp.4	male	Chongqing, Yintiaoling National Nature Reserve	OR578611

**Table 4:** Genetic distance of COX1 of *Dendrocerus* species.

	A001	A002	A003	A004	A005	A006	A007	A008	A009
A001									
A002	0.0000								
A003	0.0015	0.0015							
A004	0.0124	0.0124	0.0139						
A005	0.0000	0.0000	0.0015	0.0124					
A006	0.0000	0.0000	0.0015	0.0124	0.0000				
A007	0.1731	0.1731	0.1712	0.1849	0.1731	0.1731			
A008	0.1529	0.1529	0.1548	0.1546	0.1529	0.1529	0.1937		
A009	0.1752	0.1752	0.1772	0.1811	0.1752	0.1752	0.1715	0.2037	

## Data Availability

All sequences generated during this study have been deposited in the GenBank (https://www.ncbi.nlm.nih.gov/genbank/) (accessed on 21 September 2023).
